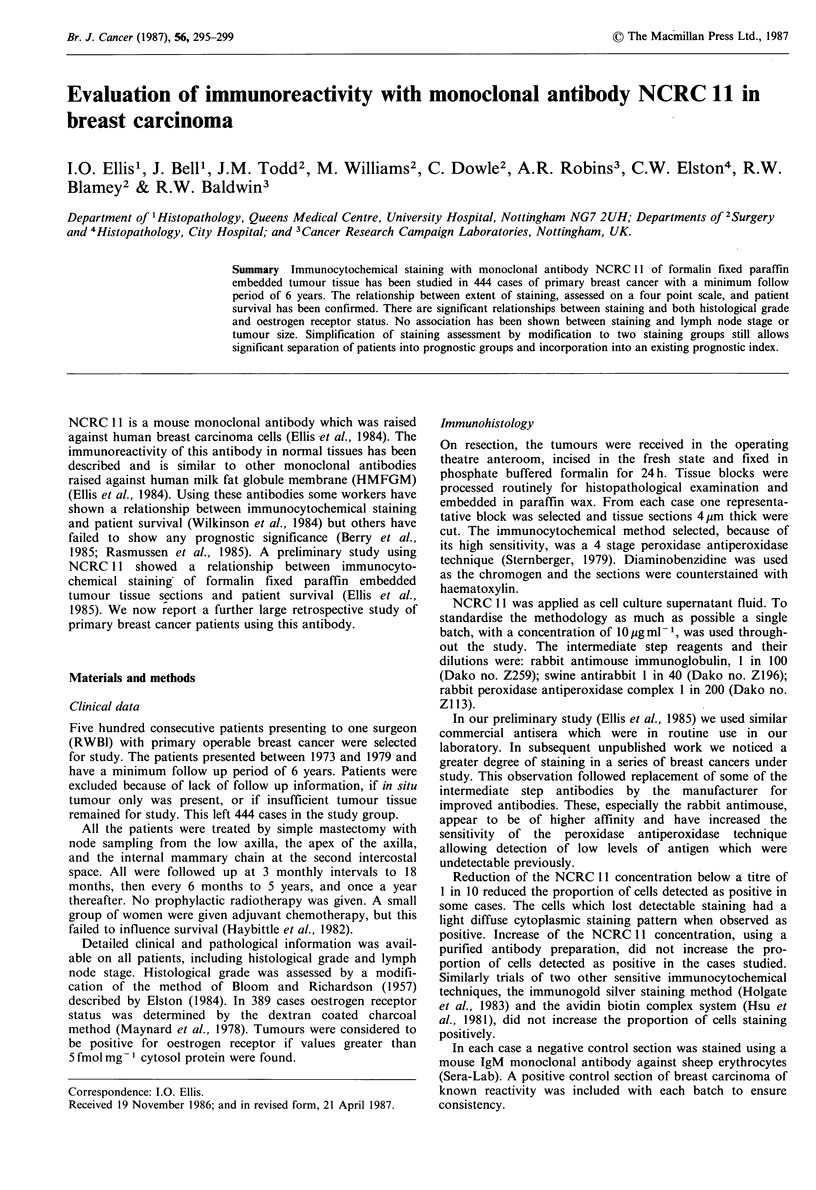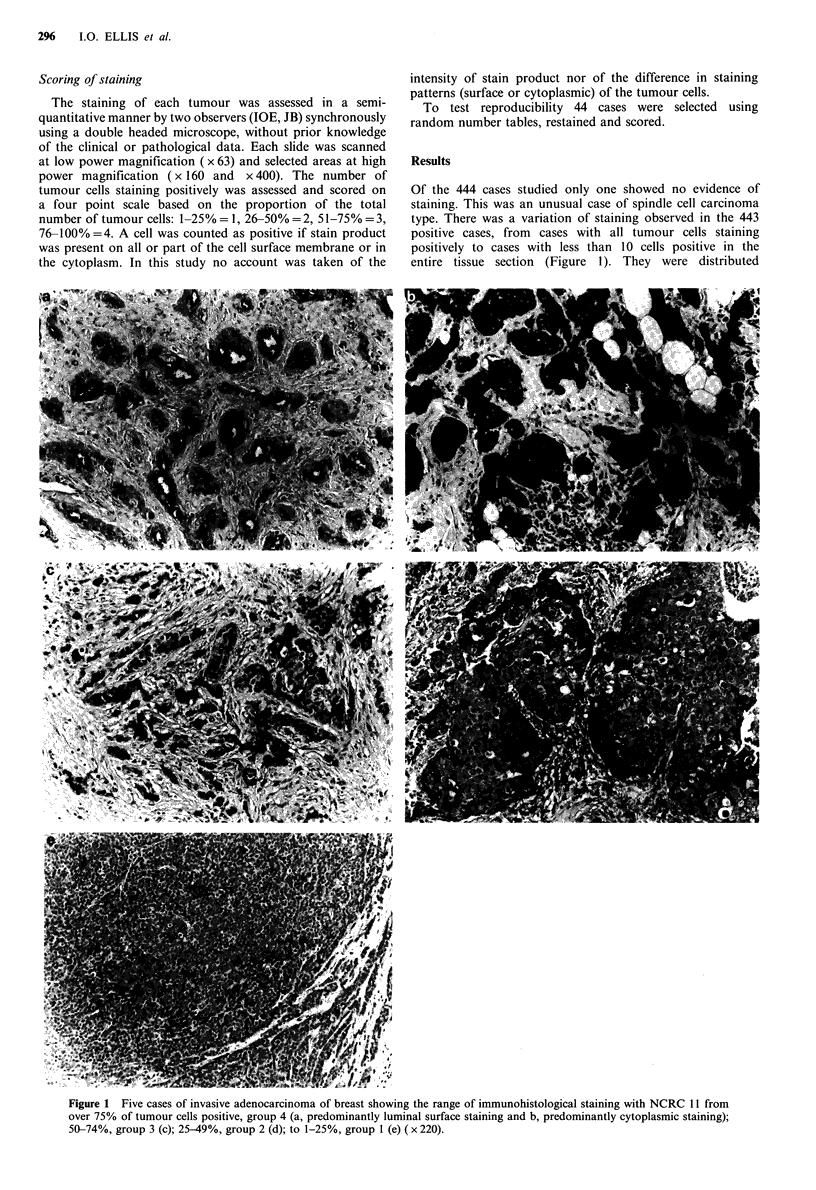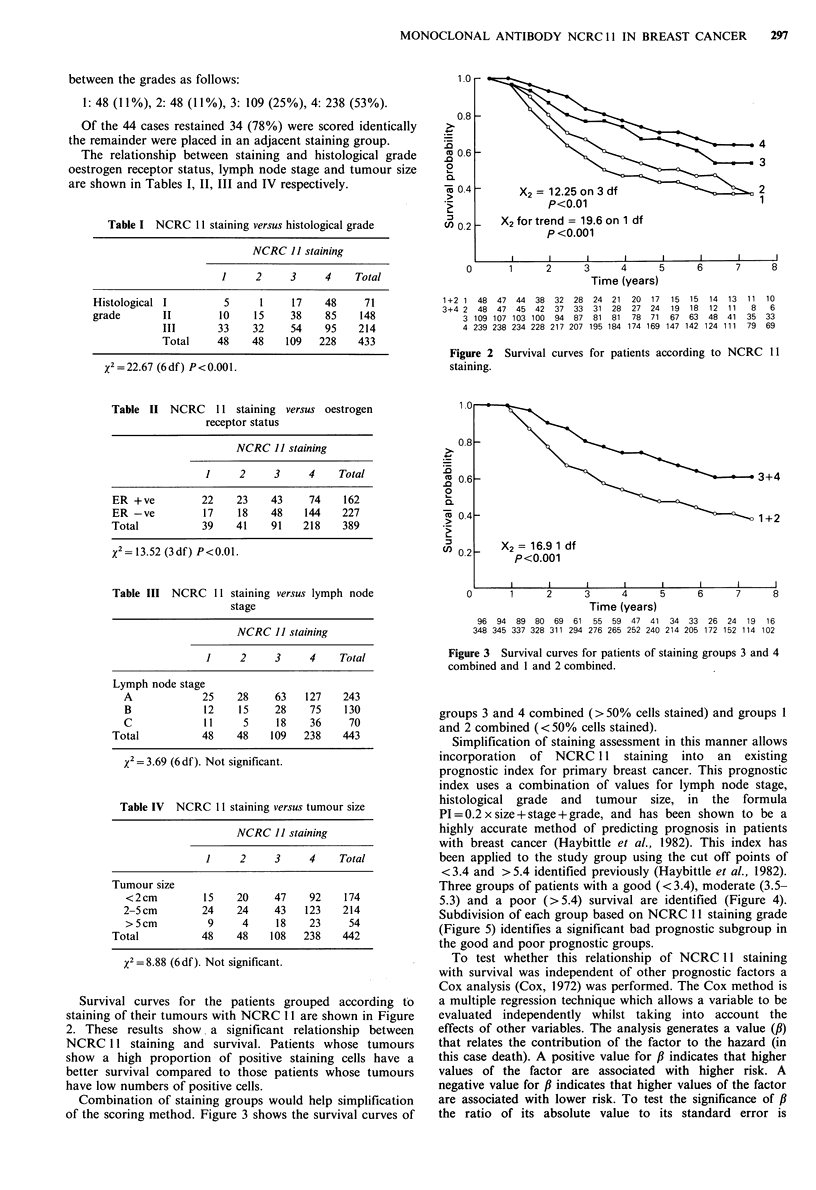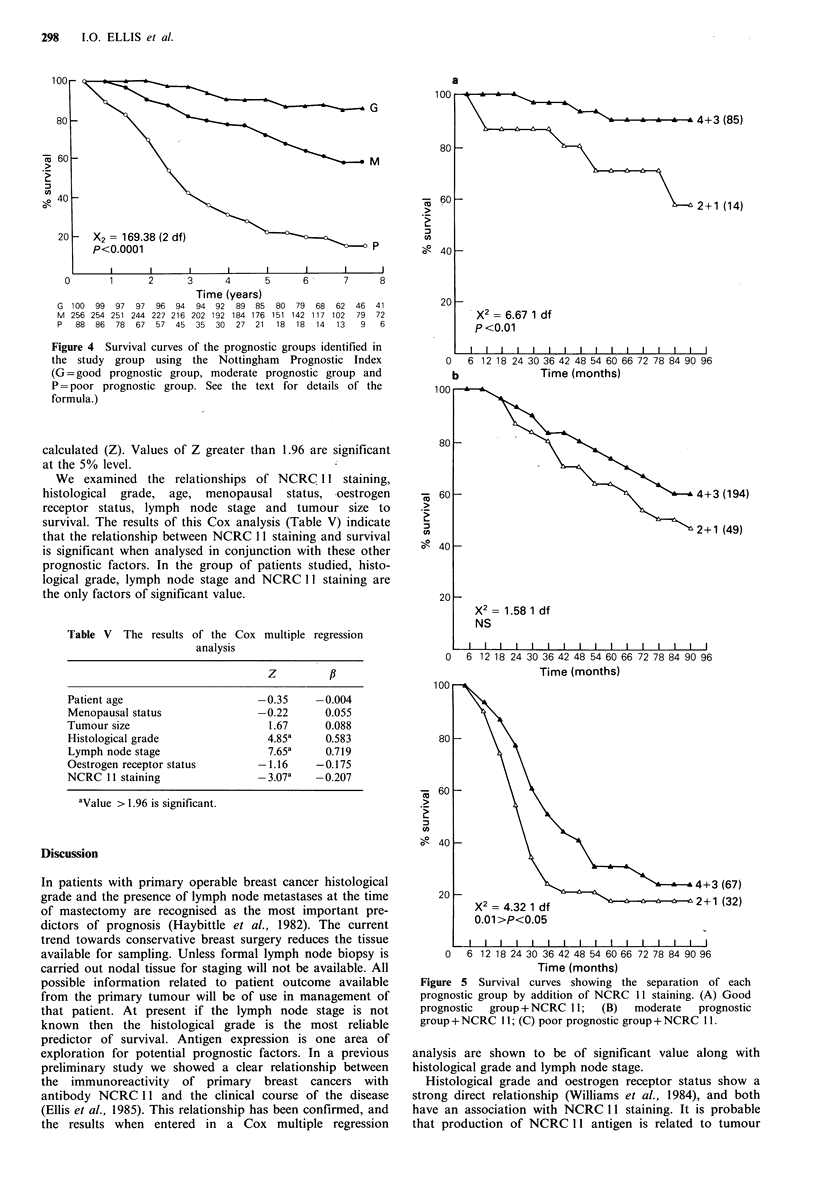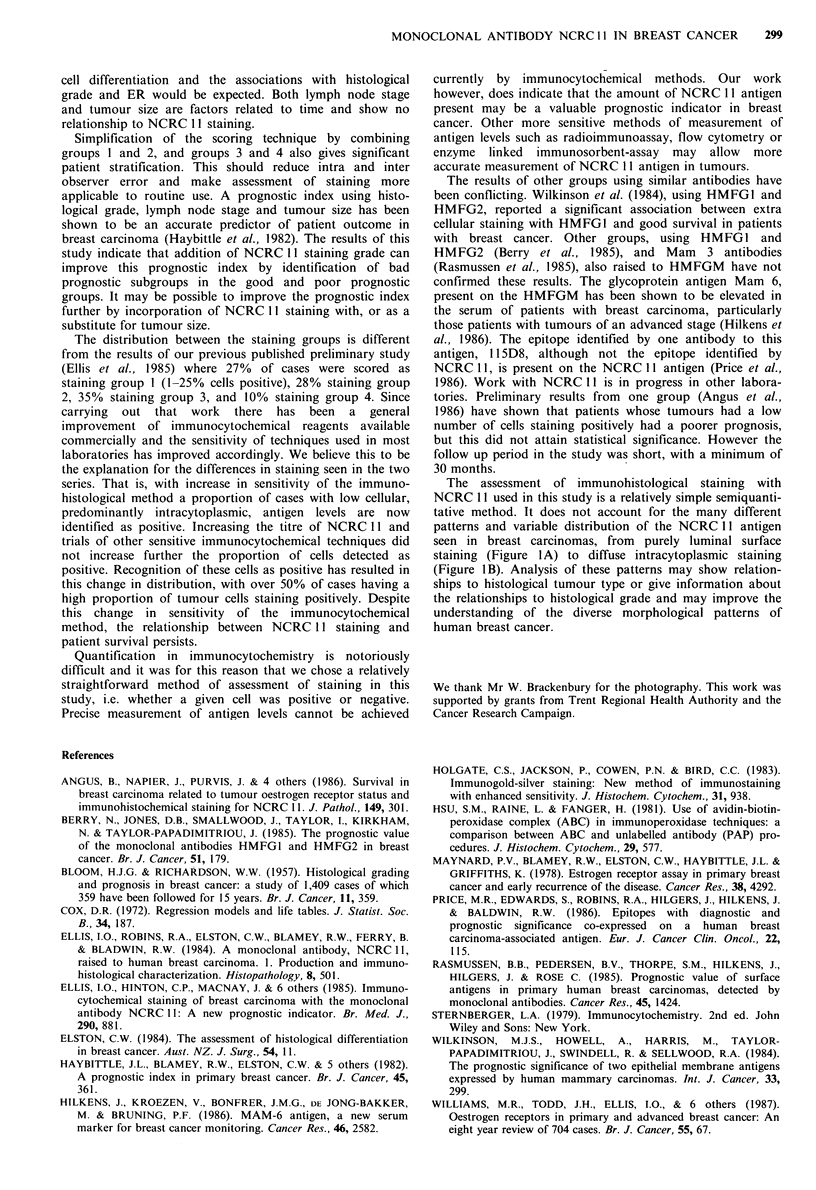# Evaluation of immunoreactivity with monoclonal antibody NCRC 11 in breast carcinoma.

**DOI:** 10.1038/bjc.1987.192

**Published:** 1987-09

**Authors:** I. O. Ellis, J. Bell, J. M. Todd, M. Williams, C. Dowle, A. R. Robins, C. W. Elston, R. W. Blamey, R. W. Baldwin

**Affiliations:** Department of Histopathology, Queens Medical Centre, University Hospital, Nottingham, UK.

## Abstract

**Images:**


*Br. J. Cancer* (1987), **56**, 295–299 © The Macmillan Press Ltd., 1987

### Evaluation of immunoreactivity with monoclonal antibody NCRC 11 in breast carcinoma

I.O. Ellis[1], J. Bell[1], J.M. Todd[2], M. Williams[2], C. Dowle[2], A.R. Robins[3], C.W. Elston[4], R.W. Blamey[2] & R.W. Baldwin[3]

*Department of [1]Histopathology, Queens Medical Centre, University Hospital, Nottingham NG7 2UH; Departments of [2]Surgery and [4]Histopathology, City Hospital; and [3]Cancer Research Campaign Laboratories, Nottingham, UK.*

**Summary** Immunocytochemical staining with monoclonal antibody NCRC 11 of formalin fixed paraffin embedded tumour tissue has been studied in 444 cases of primary breast cancer with a minimum follow period of 6 years. The relationship between extent of staining, assessed on a four point scale, and patient survival has been confirmed. There are significant relationships between staining and both histological grade and oestrogen receptor status. No association has been shown between staining and lymph node stage or tumour size. Simplification of staining assessment by modification to two staining groups still allows significant separation of patients into prognostic groups and incorporation into an existing prognostic index.

NCRC 11 is a mouse monoclonal antibody which was raised against human breast carcinoma cells (Ellis *et al.*, 1984). The immunoreactivity of this antibody in normal tissues has been described and is similar to other monoclonal antibodies raised against human milk fat globule membrane (HMFGM) (Ellis *et al.*, 1984). Using these antibodies some workers have shown a relationship between immunocytochemical staining and patient survival (Wilkinson *et al.*, 1984) but others have failed to show any prognostic significance (Berry *et al.*, 1985; Rasmussen *et al.*, 1985). A preliminary study using NCRC 11 showed a relationship between immunocytochemical staining of formalin fixed paraffin embedded tumour tissue sections and patient survival (Ellis *et al.*, 1985). We now report a further large retrospective study of primary breast cancer patients using this antibody.

### Materials and methods

### *Clinical data*

Five hundred consecutive patients presenting to one surgeon (RWBl) with primary operable breast cancer were selected for study. The patients presented between 1973 and 1979 and have a minimum follow up period of 6 years. Patients were excluded because of lack of follow up information, if *in situ* tumour only was present, or if insufficient tumour tissue remained for study. This left 444 cases in the study group.

All the patients were treated by simple mastectomy with node sampling from the low axilla, the apex of the axilla, and the internal mammary chain at the second intercostal space. All were followed up at 3 monthly intervals to 18 months, then every 6 months to 5 years, and once a year thereafter. No prophylactic radiotherapy was given. A small group of women were given adjuvant chemotherapy, but this failed to influence survival (Haybittle *et al.*, 1982).

Detailed clinical and pathological information was available on all patients, including histological grade and lymph node stage. Histological grade was assessed by a modification of the method of Bloom and Richardson (1957) described by Elston (1984). In 389 cases oestrogen receptor status was determined by the dextran coated charcoal method (Maynard *et al.*, 1978). Tumours were considered to be positive for oestrogen receptor if values greater than 5 fmol mg$^{-1}$ cytosol protein were found.

Correspondence: I.O. Ellis.
Received 19 November 1986; and in revised form, 21 April 1987.

### *Immunohistology*

On resection, the tumours were received in the operating theatre anteroom, incised in the fresh state and fixed in phosphate buffered formalin for 24 h. Tissue blocks were processed routinely for histopathological examination and embedded in paraffin wax. From each case one representatative block was selected and tissue sections 4 $\mu$m thick were cut. The immunocytochemical method selected, because of its high sensitivity, was a 4 stage peroxidase antiperoxidase technique (Sternberger, 1979). Diaminobenzidine was used as the chromogen and the sections were counterstained with haematoxylin.

NCRC 11 was applied as cell culture supernatant fluid. To standardise the methodology as much as possible a single batch, with a concentration of 10 $\mu$g ml$^{-1}$, was used throughout the study. The intermediate step reagents and their dilutions were: rabbit antimouse immunoglobulin, 1 in 100 (Dako no. Z259); swine antirabbit 1 in 40 (Dako no. Z196); rabbit peroxidase antiperoxidase complex 1 in 200 (Dako no. Z113).

In our preliminary study (Ellis *et al.*, 1985) we used similar commercial antisera which were in routine use in our laboratory. In subsequent unpublished work we noticed a greater degree of staining in a series of breast cancers under study. This observation followed replacement of some of the intermediate step antibodies by the manufacturer for improved antibodies. These, especially the rabbit antimouse, appear to be of higher affinity and have increased the sensitivity of the peroxidase antiperoxidase technique allowing detection of low levels of antigen which were undetectable previously.

Reduction of the NCRC 11 concentration below a titre of 1 in 10 reduced the proportion of cells detected as positive in some cases. The cells which lost detectable staining had a light diffuse cytoplasmic staining pattern when observed as positive. Increase of the NCRC 11 concentration, using a purified antibody preparation, did not increase the proportion of cells detected as positive in the cases studied. Similarly trials of two other sensitive immunocytochemical techniques, the immunogold silver staining method (Holgate *et al.*, 1983) and the avidin biotin complex system (Hsu *et al.*, 1981), did not increase the proportion of cells staining positively.

In each case a negative control section was stained using a mouse IgM monoclonal antibody against sheep erythrocytes (Sera-Lab). A positive control section of breast carcinoma of known reactivity was included with each batch to ensure consistency.

*Scoring of staining*

The staining of each tumour was assessed in a semi-quantitative manner by two observers (IOE, JB) synchronously using a double headed microscope, without prior knowledge of the clinical or pathological data. Each slide was scanned at low power magnification ( × 63) and selected areas at high power magnification ( × 160 and × 400). The number of tumour cells staining positively was assessed and scored on a four point scale based on the proportion of the total number of tumour cells: 1–25% = 1, 26–50% = 2, 51–75% = 3, 76–100% = 4. A cell was counted as positive if stain product was present on all or part of the cell surface membrane or in the cytoplasm. In this study no account was taken of the intensity of stain product nor of the difference in staining patterns (surface or cytoplasmic) of the tumour cells.

To test reproducibility 44 cases were selected using random number tables, restained and scored.

### Results

Of the 444 cases studied only one showed no evidence of staining. This was an unusual case of spindle cell carcinoma type. There was a variation of staining observed in the 443 positive cases, from cases with all tumour cells staining positively to cases with less than 10 cells positive in the entire tissue section (Figure 1). They were distributed

**Figure 1** Five cases of invasive adenocarcinoma of breast showing the range of immunohistological staining with NCRC 11 from over 75% of tumour cells positive, group 4 (a, predominantly luminal surface staining and b, predominantly cytoplasmic staining); 50–74%, group 3 (c); 25–49%, group 2 (d); to 1–25%, group 1 (e) ( × 220).

between the grades as follows:

1: 48 (11%), 2: 48 (11%), 3: 109 (25%), 4: 238 (53%).

Of the 44 cases restained 34 (78%) were scored identically the remainder were placed in an adjacent staining group.

The relationship between staining and histological grade oestrogen receptor status, lymph node stage and tumour size are shown in Tables I, II, III and IV respectively.

**Table I** NCRC 11 staining *versus* histological grade

| | | *NCRC 11 staining* | | | | |
|---|---|---|---|---|---|---|
| | | *1* | *2* | *3* | *4* | *Total* |
| Histological grade | I | 5 | 1 | 17 | 48 | 71 |
| | II | 10 | 15 | 38 | 85 | 148 |
| | III | 33 | 32 | 54 | 95 | 214 |
| | Total | 48 | 48 | 109 | 228 | 433 |

$\chi^2 = 22.67$ (6 df) $P < 0.001$.

**Table II** NCRC 11 staining *versus* oestrogen receptor status

| | *NCRC 11 staining* | | | | |
|---|---|---|---|---|---|
| | *1* | *2* | *3* | *4* | *Total* |
| ER +ve | 22 | 23 | 43 | 74 | 162 |
| ER −ve | 17 | 18 | 48 | 144 | 227 |
| Total | 39 | 41 | 91 | 218 | 389 |

$\chi^2 = 13.52$ (3 df) $P < 0.01$.

**Table III** NCRC 11 staining *versus* lymph node stage

| | *NCRC 11 staining* | | | | |
|---|---|---|---|---|---|
| | *1* | *2* | *3* | *4* | *Total* |
| Lymph node stage | | | | | |
| A | 25 | 28 | 63 | 127 | 243 |
| B | 12 | 15 | 28 | 75 | 130 |
| C | 11 | 5 | 18 | 36 | 70 |
| Total | 48 | 48 | 109 | 238 | 443 |

$\chi^2 = 3.69$ (6 df). Not significant.

**Table IV** NCRC 11 staining *versus* tumour size

| | *NCRC 11 staining* | | | | |
|---|---|---|---|---|---|
| | *1* | *2* | *3* | *4* | *Total* |
| Tumour size | | | | | |
| <2 cm | 15 | 20 | 47 | 92 | 174 |
| 2–5 cm | 24 | 24 | 43 | 123 | 214 |
| >5 cm | 9 | 4 | 18 | 23 | 54 |
| Total | 48 | 48 | 108 | 238 | 442 |

$\chi^2 = 8.88$ (6 df). Not significant.

Survival curves for the patients grouped according to staining of their tumours with NCRC 11 are shown in Figure 2. These results show a significant relationship between NCRC 11 staining and survival. Patients whose tumours show a high proportion of positive staining cells have a better survival compared to those patients whose tumours have low numbers of positive cells.

Combination of staining groups would help simplification of the scoring method. Figure 3 shows the survival curves of

**Figure 2** Survival curves for patients according to NCRC 11 staining.

**Figure 3** Survival curves for patients of staining groups 3 and 4 combined and 1 and 2 combined.

groups 3 and 4 combined (>50% cells stained) and groups 1 and 2 combined (<50% cells stained).

Simplification of staining assessment in this manner allows incorporation of NCRC 11 staining into an existing prognostic index for primary breast cancer. This prognostic index uses a combination of values for lymph node stage, histological grade and tumour size, in the formula $PI = 0.2 \times \text{size} + \text{stage} + \text{grade}$, and has been shown to be a highly accurate method of predicting prognosis in patients with breast cancer (Haybittle *et al.*, 1982). This index has been applied to the study group using the cut off points of <3.4 and >5.4 identified previously (Haybittle *et al.*, 1982). Three groups of patients with a good (<3.4), moderate (3.5–5.3) and a poor (>5.4) survival are identified (Figure 4). Subdivision of each group based on NCRC 11 staining grade (Figure 5) identifies a significant bad prognostic subgroup in the good and poor prognostic groups.

To test whether this relationship of NCRC 11 staining with survival was independent of other prognostic factors a Cox analysis (Cox, 1972) was performed. The Cox method is a multiple regression technique which allows a variable to be evaluated independently whilst taking into account the effects of other variables. The analysis generates a value ($\beta$) that relates the contribution of the factor to the hazard (in this case death). A positive value for $\beta$ indicates that higher values of the factor are associated with higher risk. A negative value for $\beta$ indicates that higher values of the factor are associated with lower risk. To test the significance of $\beta$ the ratio of its absolute value to its standard error is

| | | | | | | | | | | | | | | | | |
|---|---|---|---|---|---|---|---|---|---|---|---|---|---|---|---|---|
| G | 100 | 99 | 97 | 97 | 96 | 94 | 94 | 92 | 89 | 85 | 80 | 79 | 68 | 62 | 46 | 41 |
| M | 256 | 254 | 251 | 244 | 227 | 216 | 202 | 192 | 184 | 176 | 151 | 142 | 117 | 102 | 79 | 72 |
| P | 88 | 86 | 78 | 67 | 57 | 45 | 35 | 30 | 27 | 21 | 18 | 18 | 14 | 13 | 9 | 6 |

**Figure 4** Survival curves of the prognostic groups identified in the study group using the Nottingham Prognostic Index (G = good prognostic group, moderate prognostic group and P = poor prognostic group. See the text for details of the formula.)

calculated (Z). Values of Z greater than 1.96 are significant at the 5% level.

We examined the relationships of NCRC 11 staining, histological grade, age, menopausal status, oestrogen receptor status, lymph node stage and tumour size to survival. The results of this Cox analysis (Table V) indicate that the relationship between NCRC 11 staining and survival is significant when analysed in conjunction with these other prognostic factors. In the group of patients studied, histological grade, lymph node stage and NCRC 11 staining are the only factors of significant value.

**Table V** The results of the Cox multiple regression analysis

| | *Z* | *β* |
|---|---|---|
| Patient age | −0.35 | −0.004 |
| Menopausal status | −0.22 | 0.055 |
| Tumour size | 1.67 | 0.088 |
| Histological grade | 4.85[a] | 0.583 |
| Lymph node stage | 7.65[a] | 0.719 |
| Oestrogen receptor status | −1.16 | −0.175 |
| NCRC 11 staining | −3.07[a] | −0.207 |

[a]Value > 1.96 is significant.

### Discussion

In patients with primary operable breast cancer histological grade and the presence of lymph node metastases at the time of mastectomy are recognised as the most important predictors of prognosis (Haybittle *et al.*, 1982). The current trend towards conservative breast surgery reduces the tissue available for sampling. Unless formal lymph node biopsy is carried out nodal tissue for staging will not be available. All possible information related to patient outcome available from the primary tumour will be of use in management of that patient. At present if the lymph node stage is not known then the histological grade is the most reliable predictor of survival. Antigen expression is one area of exploration for potential prognostic factors. In a previous preliminary study we showed a clear relationship between the immunoreactivity of primary breast cancers with antibody NCRC 11 and the clinical course of the disease (Ellis *et al.*, 1985). This relationship has been confirmed, and the results when entered in a Cox multiple regression analysis are shown to be of significant value along with histological grade and lymph node stage.

**Figure 5** Survival curves showing the separation of each prognostic group by addition of NCRC 11 staining. (A) Good prognostic group + NCRC 11; (B) moderate prognostic group + NCRC 11; (C) poor prognostic group + NCRC 11.

Histological grade and oestrogen receptor status show a strong direct relationship (Williams *et al.*, 1984), and both have an association with NCRC 11 staining. It is probable that production of NCRC 11 antigen is related to tumour cell differentiation and the associations with histological grade and ER would be expected. Both lymph node stage and tumour size are factors related to time and show no relationship to NCRC 11 staining.

Simplification of the scoring technique by combining groups 1 and 2, and groups 3 and 4 also gives significant patient stratification. This should reduce intra and inter observer error and make assessment of staining more applicable to routine use. A prognostic index using histological grade, lymph node stage and tumour size has been shown to be an accurate predictor of patient outcome in breast carcinoma (Haybittle *et al.*, 1982). The results of this study indicate that addition of NCRC 11 staining grade can improve this prognostic index by identification of bad prognostic subgroups in the good and poor prognostic groups. It may be possible to improve the prognostic index further by incorporation of NCRC 11 staining with, or as a substitute for tumour size.

The distribution between the staining groups is different from the results of our previous published preliminary study (Ellis *et al.*, 1985) where 27% of cases were scored as staining group 1 (1–25% cells positive), 28% staining group 2, 35% staining group 3, and 10% staining group 4. Since carrying out that work there has been a general improvement of immunocytochemical reagents available commercially and the sensitivity of techniques used in most laboratories has improved accordingly. We believe this to be the explanation for the differences in staining seen in the two series. That is, with increase in sensitivity of the immunohistological method a proportion of cases with low cellular, predominantly intracytoplasmic, antigen levels are now identified as positive. Increasing the titre of NCRC 11 and trials of other sensitive immunocytochemical techniques did not increase further the proportion of cells detected as positive. Recognition of these cells as positive has resulted in this change in distribution, with over 50% of cases having a high proportion of tumour cells staining positively. Despite this change in sensitivity of the immunocytochemical method, the relationship between NCRC 11 staining and patient survival persists.

Quantification in immunocytochemistry is notoriously difficult and it was for this reason that we chose a relatively straightforward method of assessment of staining in this study, i.e. whether a given cell was positive or negative. Precise measurement of antigen levels cannot be achieved currently by immunocytochemical methods. Our work however, does indicate that the amount of NCRC 11 antigen present may be a valuable prognostic indicator in breast cancer. Other more sensitive methods of measurement of antigen levels such as radioimmunoassay, flow cytometry or enzyme linked immunosorbent-assay may allow more accurate measurement of NCRC 11 antigen in tumours.

The results of other groups using similar antibodies have been conflicting. Wilkinson *et al.* (1984), using HMFG1 and HMFG2, reported a significant association between extra cellular staining with HMFG1 and good survival in patients with breast cancer. Other groups, using HMFG1 and HMFG2 (Berry *et al.*, 1985), and Mam 3 antibodies (Rasmussen *et al.*, 1985), also raised to HMFGM have not confirmed these results. The glycoprotein antigen Mam 6, present on the HMFGM has been shown to be elevated in the serum of patients with breast carcinoma, particularly those patients with tumours of an advanced stage (Hilkens *et al.*, 1986). The epitope identified by one antibody to this antigen, 115D8, although not the epitope identified by NCRC 11, is present on the NCRC 11 antigen (Price *et al.*, 1986). Work with NCRC 11 is in progress in other laboratories. Preliminary results from one group (Angus *et al.*, 1986) have shown that patients whose tumours had a low number of cells staining positively had a poorer prognosis, but this did not attain statistical significance. However the follow up period in the study was short, with a minimum of 30 months.

The assessment of immunohistological staining with NCRC 11 used in this study is a relatively simple semiquantitative method. It does not account for the many different patterns and variable distribution of the NCRC 11 antigen seen in breast carcinomas, from purely luminal surface staining (Figure 1A) to diffuse intracytoplasmic staining (Figure 1B). Analysis of these patterns may show relationships to histological tumour type or give information about the relationships to histological grade and may improve the understanding of the diverse morphological patterns of human breast cancer.

We thank Mr W. Brackenbury for the photography. This work was supported by grants from Trent Regional Health Authority and the Cancer Research Campaign.

### References

ANGUS, B., NAPIER, J., PURVIS, J. & 4 others (1986). Survival in breast carcinoma related to tumour oestrogen receptor status and immunohistochemical staining for NCRC 11. *J. Pathol.*, **149**, 301.

BERRY, N., JONES, D.B., SMALLWOOD, J., TAYLOR, I., KIRKHAM, N. & TAYLOR-PAPADIMITRIOU, J. (1985). The prognostic value of the monoclonal antibodies HMFG1 and HMFG2 in breast cancer. *Br. J. Cancer*, **51**, 179.

BLOOM, H.J.G. & RICHARDSON, W.W. (1957). Histological grading and prognosis in breast cancer: a study of 1,409 cases of which 359 have been followed for 15 years. *Br. J. Cancer*, **11**, 359.

COX, D.R. (1972). Regression models and life tables. *J. Statist. Soc. B.*, **34**, 187.

ELLIS, I.O., ROBINS, R.A., ELSTON, C.W., BLAMEY, R.W., FERRY, B. & BLADWIN, R.W. (1984). A monoclonal antibody, NCRC 11, raised to human breast carcinoma. 1. Production and immunohistological characterization. *Histopathology*, **8**, 501.

ELLIS, I.O., HINTON, C.P., MACNAY, J. & 6 others (1985). Immunocytochemical staining of breast carcinoma with the monoclonal antibody NCRC 11: A new prognostic indicator. *Br. Med. J.*, **290**, 881.

ELSTON, C.W. (1984). The assessment of histological differentiation in breast cancer. *Aust. NZ. J. Surg.*, **54**, 11.

HAYBITTLE, J.L., BLAMEY, R.W., ELSTON, C.W. & 5 others (1982). A prognostic index in primary breast cancer. *Br. J. Cancer*, **45**, 361.

HILKENS, J., KROEZEN, V., BONFRER, J.M.G., DE JONG-BAKKER, M. & BRUNING, P.F. (1986). MAM-6 antigen, a new serum marker for breast cancer monitoring. *Cancer Res.*, **46**, 2582.

HOLGATE, C.S., JACKSON, P., COWEN, P.N. & BIRD, C.C. (1983). Immunogold-silver staining: New method of immunostaining with enhanced sensitivity. *J. Histochem. Cytochem.*, **31**, 938.

HSU, S.M., RAINE, L. & FANGER, H. (1981). Use of avidin-biotin-peroxidase complex (ABC) in immunoperoxidase techniques: a comparison between ABC and unlabelled antibody (PAP) procedures. *J. Histochem. Cytochem.*, **29**, 577.

MAYNARD, P.V., BLAMEY, R.W., ELSTON, C.W., HAYBITTLE, J.L. & GRIFFITHS, K. (1978). Estrogen receptor assay in primary breast cancer and early recurrence of the disease. *Cancer Res.*, **38**, 4292.

PRICE, M.R., EDWARDS, S., ROBINS, R.A., HILGERS, J., HILKENS, J. & BALDWIN, R.W. (1986). Epitopes with diagnostic and prognostic significance co-expressed on a human breast carcinoma-associated antigen. *Eur. J. Cancer Clin. Oncol.*, **22**, 115.

RASMUSSEN, B.B., PEDERSEN, B.V., THORPE, S.M., HILKENS, J., HILGERS, J. & ROSE C. (1985). Prognostic value of surface antigens in primary human breast carcinomas, detected by monoclonal antibodies. *Cancer Res.*, **45**, 1424.

STERNBERGER, L.A. (1979). Immunocytochemistry. 2nd ed. John Wiley and Sons: New York.

WILKINSON, M.J.S., HOWELL, A., HARRIS, M., TAYLOR-PAPADIMITRIOU, J., SWINDELL, R. & SELLWOOD, R.A. (1984). The prognostic significance of two epithelial membrane antigens expressed by human mammary carcinomas. *Int. J. Cancer*, **33**, 299.

WILLIAMS, M.R., TODD, J.H., ELLIS, I.O., & 6 others (1987). Oestrogen receptors in primary and advanced breast cancer: An eight year review of 704 cases. *Br. J. Cancer*, **55**, 67.